# Supplementary material for: Regulatory mechanism predates the evolution of self-organizing capacity in simulated ant-like robots
Source: Commun Biol. 2019 Jan 18;2:25. doi: 10.1038/s42003-018-0276-3 (PMC6338667; doi:10.1038/s42003-018-0276-3)
Supplement: Supplementary file 1 — Supplementary Information [file 42003_2018_276_MOESM1_ESM.pdf]

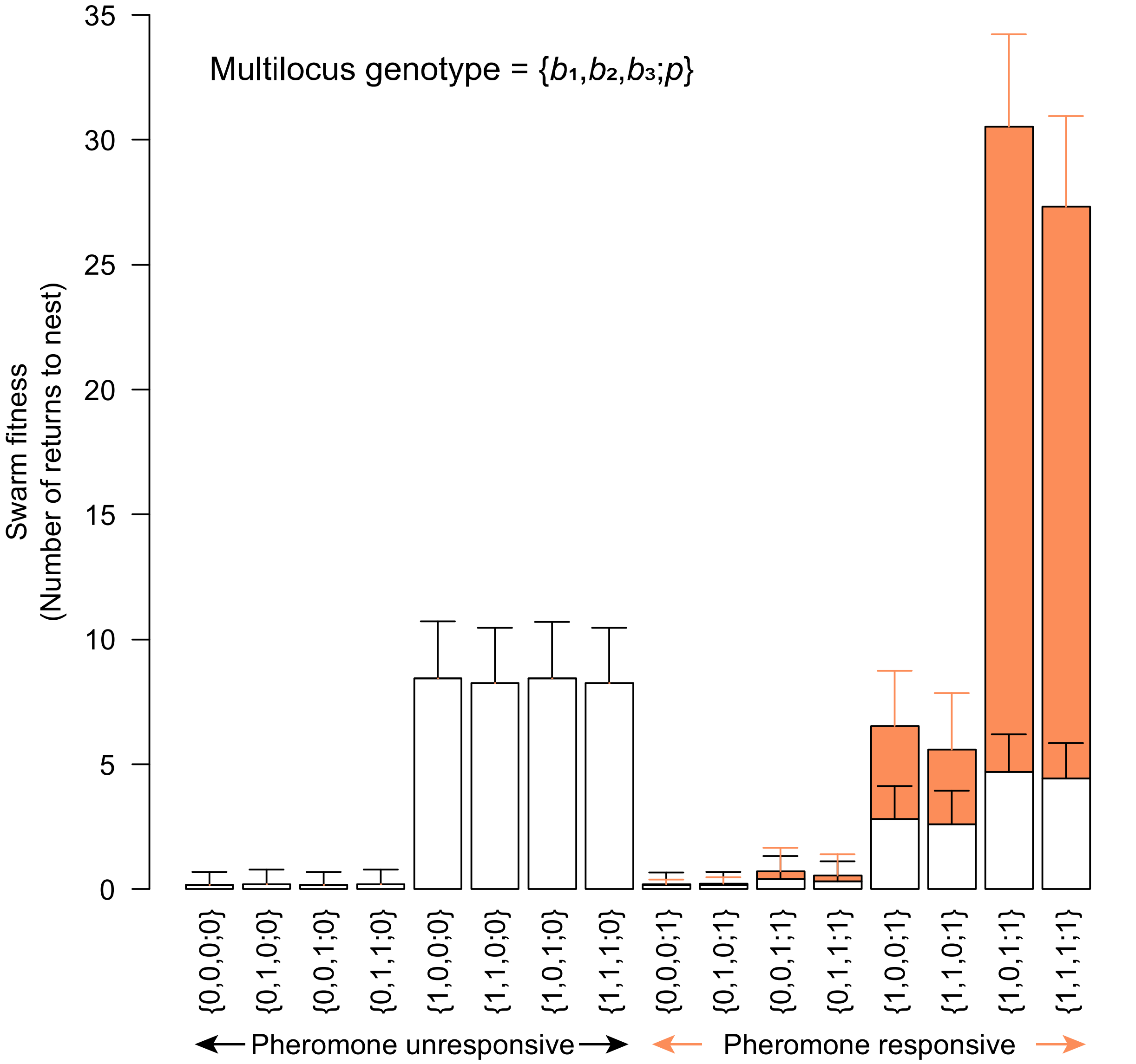

Supplementary Figure 1. Measures of group foraging performance, highlighting the effect of pheromone communication. The source data are the same as Fig. 2. Total number of foraging bouts per swarm is a measure of swarm fitness. Bars indicate that the food was found by robots in state S_1 (white, searching independently) or S_3 (orange, being recruited). Error bars show SD (n = 100 000 trials each).

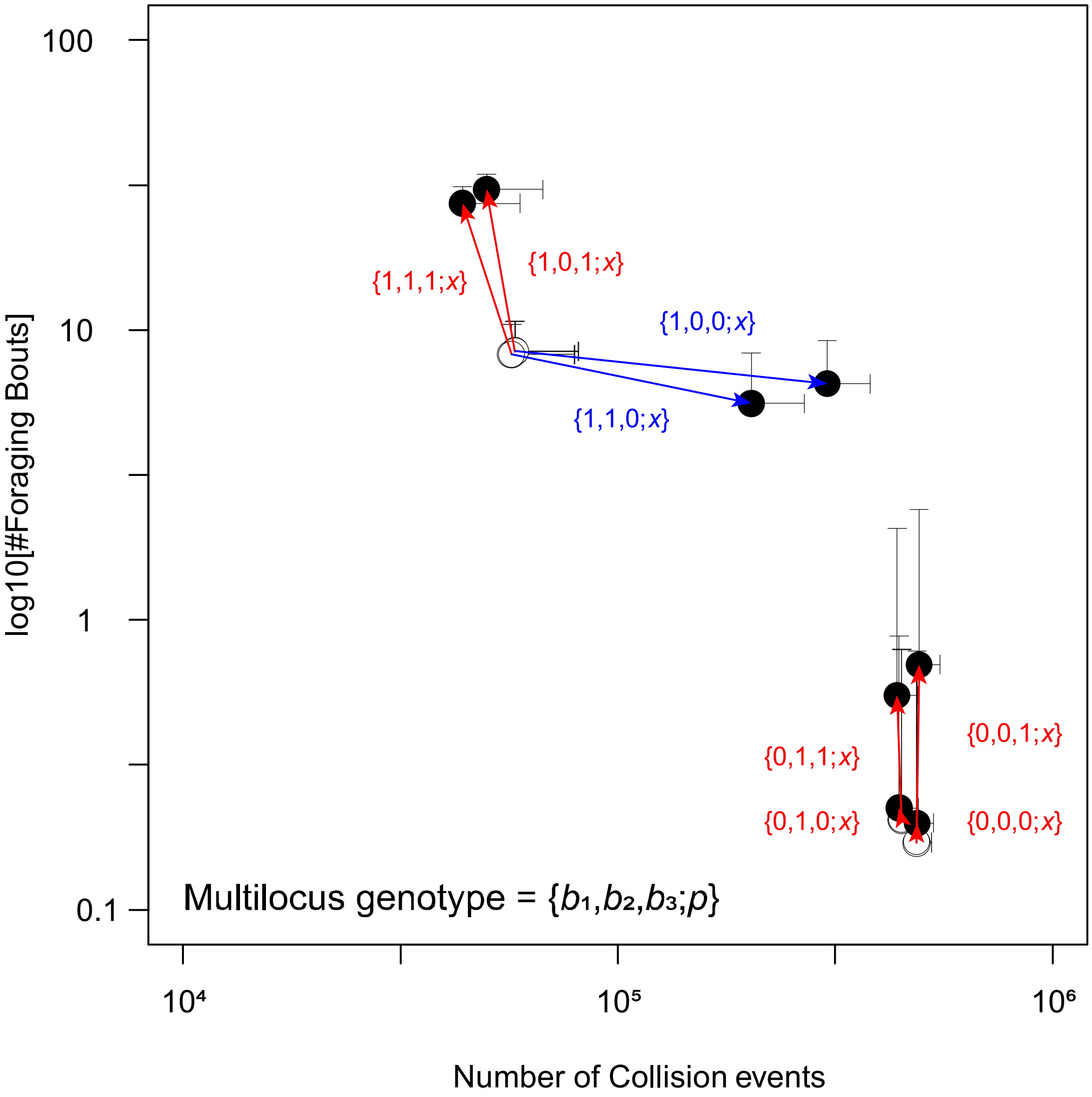

Supplementary Figure 2. Relationship between total number of collisions and group foraging performance. Each circle represents a genotype, with error bars showing SD: ○ absence ($p = 0$), ● presence ($p = 1$) of the pheromone responsiveness trait. Arrows connect genotypes with the same behavioral traits (b_1, b_2, and b_3), making clear the varying effects of pheromone communication on group foraging performance. The particular combinations of behavioral genotypes $\{b_1, b_2, b_3\}$ (red arrows; including = {1, 0, 1}) made pheromone communication improve the group foraging performance by reducing the occurrence of collisions, while the pheromone communication caused the other behavioral genotypes (blue arrows) to reduce the efficiency of group foraging through increased collisions.

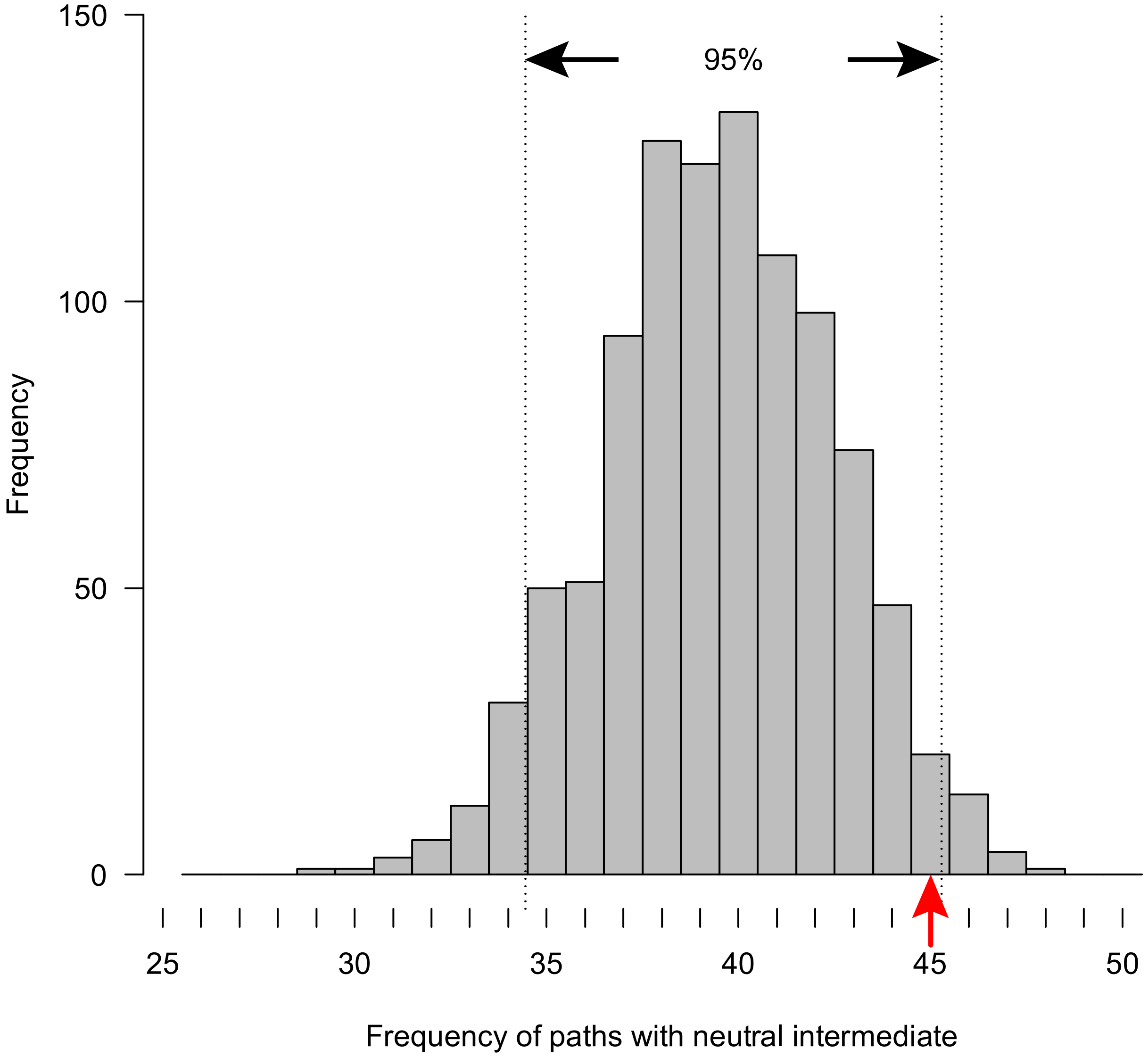

Supplementary Figure 3. Resampled distribution of the number of populations (out of 50) that take the neutral intermediate genotype {1,0,1;0}. The observed frequency (45, red arrow) was placed at the borderline of the 95% interval between the 2.5th (34) and 97.5th (45) percentiles.

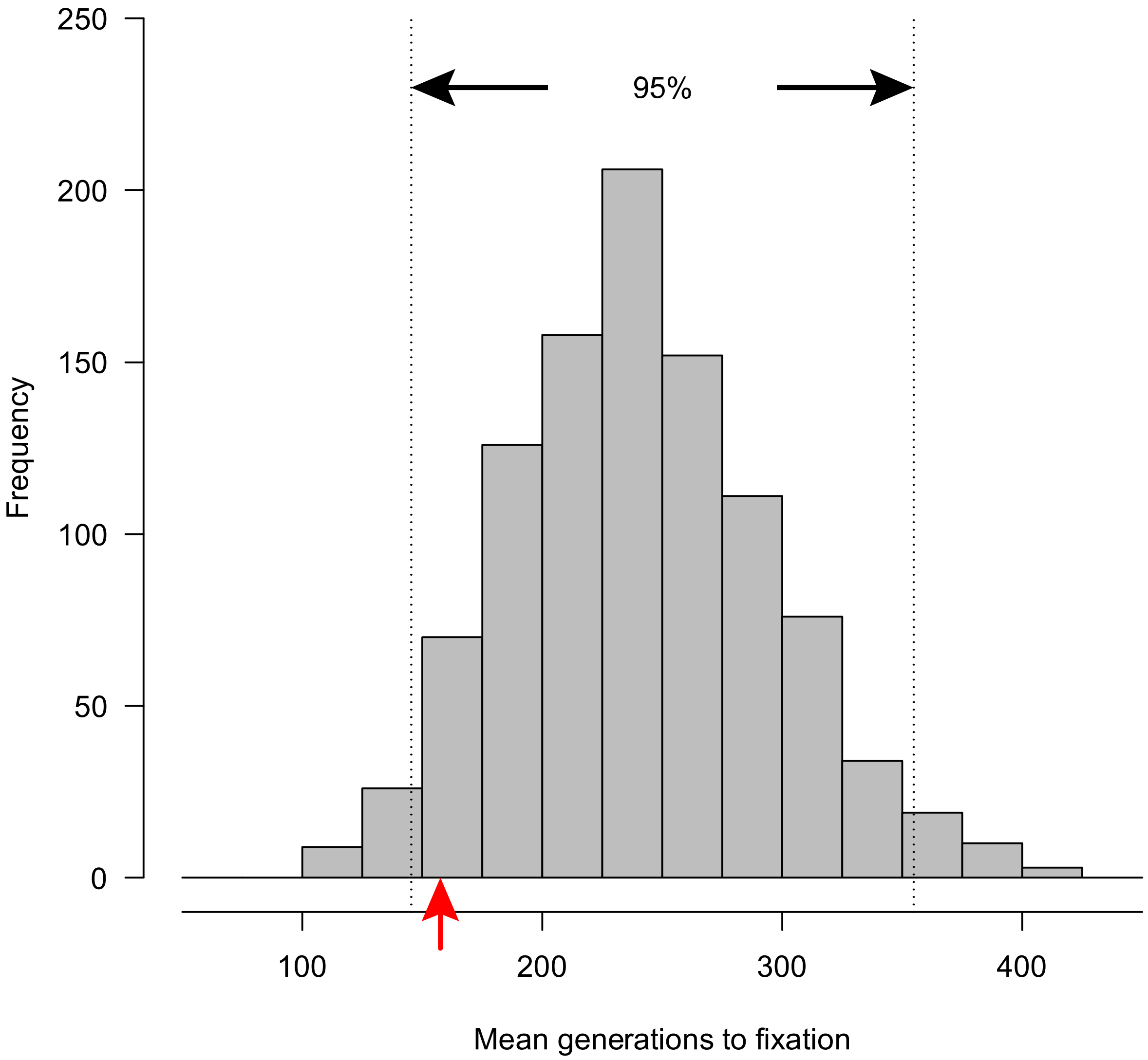

Supplementary Figure 4. Resampled distribution of the mean time (number of generations) to fixation of the final genotype {1,0,1;1}, starting from the population fixed at the original genotype {1,0,0;0}. The observed mean time to fixation (157.68 generations) fell within the 95% interval between the 2.5th (145.54) and 97.5th (354.76) percentiles.

Supplementary Table 1. Phenotypes of the 16 multilocus genotypes.

**Multilocus genotype**	b_1	b_2	b_3	p	**Traffic rule on the trail** [a]	**Traffic rule off the trail**	**Pheromone communication**
{0,0,0;0}	0	0	0	0	(Both stay)	Both stay	No
{0,1,0;0}	0	1	0	0	(Outbound first)		
{0,0,1;0}	0	0	1	0	(Inbound first)		
{0,1,1;0}	0	1	1	0	(Both give way)		
**{1,0,0;0}**	1	0	0	0	(Both stay)	Both give way	
{1,1,0;0}	1	1	0	0	(Outbound first)		
**{1,0,1;0}**	1	0	1	0	(Inbound first)		
{1,1,1;0}	1	1	1	0	(Both give way)		
{0,0,0;1}	0	0	0	1	Both stay	Both stay	Yes
{0,1,0;1}	0	1	0	1	Outbound first		
{0,0,1;1}	0	0	1	1	Inbound first		
{0,1,1;1}	0	1	1	1	Both give way		
**{1,0,0;1}**	1	0	0	1	Both stay	Both give way	
{1,1,0;1}	1	1	0	1	Outbound first		
**{1,0,1;1}**	1	0	1	1	Inbound first		
{1,1,1;1}	1	1	1	1	Both give way		

a, On the pheromone trail, a robot with behavior 'Stay' was considered to take a priority over the collision partner with behavior 'Leave.' The rules in parentheses denote that the phenotypes are cryptic in the absence of pheromone communication.

Supplementary Table 2. Summary of the results of evolutionary simulation runs.

Run_ID	Generation: {1,0,0;0} fixed	Generation: {1,0,1;1} arose	Frequency of precursor	Evolutionary path[c]	Generation: {1,0,1;1} fixed	Generations to fixation
doba-01	21	89	0.165	N	97	76
doba-02	4	82	0.125	N	97	93
doba-03	74	176	0.08	N	185	111
doba-04	14	50	0.025	N	70	56
doba-05	30	111	0.12	N	122	92
doba-06	26	201	0.005	N	211	185
doba-07	4 (0.985)[a]	35	0.215	N	63	59
doba-08	13 (0.99)[a]	147	0.25	N	158	145
doba-09	40	127	0.035	N	136	96
fuji03-01	9	28	0.095	N	38	29
fuji03-02	18	108	0.08	N	134	116
fuji03-03	4 (0.99)[a]	261	0.06	N	281	277
fuji03-04	7	64	0.01	N	75	68
fuji03-05	28	77	0.045	N	86	58
fuji03-06	7	23	0.02	N	34	27
fuji03-07	4 (0.985)[a]	117	0.11	N	126	122
fuji03-08	13	77	0.12	N	105	92
fuji03-09	*30*	*65*	*0.01*	*V*	*101*	*71*
fuji03-10	11	201	0.035	N	211	200
fuji03-11	*19*	*166*	*0.015*	*V*	*181*	*162*
fuji03-12	18	97	0.09	N	119	101
fuji03-13	15	80	0.2	N	90	75
fuji03-14	22	116	0.195	N	125	103
fuji03-15	10	103	0.13	N	116	106
fuji03-16	6	244	0.175	N	258	252
fuji04-01	6 (0.99)[a]	21	0.095	N	30	24
fuji04-02	*76*	*181*	*0.015*	*V*	*197*	*121*
fuji04-03	7	680	0.095	N	688	681
fuji04-04	14	641	0.2	N	662	648
fuji04-05	4	123	0.005	N	134	130
fuji04-06	14	365	0.305	N	394	380
fuji04-07	11	131	0.03	N	143	132
fuji04-08	13	101	0.365	N	109	96
fuji04-09	9	148	0.015	N	159	150
fuji04-10	*7*	*57*	*0.015*	*V*	*79*	*72*
fuji04-11	15 (0.995)[a]	21	0.005	N	51	36
fuji04-12	39 (0.99)[a]	217	0.36	N	262	223
fuji04-13	12	322	0.005	N	331	319
fuji04-14	8	198	0.005[b]	O	261[d]	253[d]
fuji04-15	14	67	0.075	N	76	62
ichi-01	8	130	0.15	N	140	132
ichi-02	*11*	*262*	*0.02*	*V*	*271*	*260*
ichi-03	55	157	0.145	N	182	127
ichi-04	91	227	0.16	N	236	145
ichi-05	25	83	0.09	N	92	67
ichi-06	5	113	0.21	N	123	118
ichi-07	5	247	0.21	N	259	254
ichi-08	64	428	0.245	N	447	383
ichi-09	6	130	0.685	N	142	136
ichi-10	6	159	0.16	N	169	163

a, Not fixed at {1,0,0;0}. The generation with the highest frequency (shown in the parenthesis) was taken instead.
b, The frequency of {1,1,1;0} (a precursor of {1,1,1;1}) was shown instead
c, N: neutral path with {1,0,1;0}; V: inferior path (fitness valley) with {1,0,0;1}; O, other path (see the main text).
d, The time to fixation of {1,1,1;1} was shown instead

Supplementary Note 1. Population genetic model of stochastic tunneling

In this note, we describe a detail of the population genetic analysis of stochastic tunneling. We follow Proulx's formula (1) to calculate v_1 (the probability of extinction of the final genotype lineage arising from a single swarm of the intermediate genotype with relative swarm fitness r_x; $x \in \{0, -\}$) for Wright–Fisher populations. In the Wright–Fisher formulation, unlike the Moran model, the population at generation $t + 1$ is produced from the population at generation t all at once. The probability distribution of an intermediate genotype at generation $t + 1$ is given by the following binomial distribution:

$$\Pr(0 \to 0) = 1$$

$$\Pr(i \to j) = \binom{N}{j} \left(\frac{ir}{ir+(N-i)}\right)^j \left(1 - \frac{ir}{ir+(N-i)}\right)^{N-j}$$

$$\Pr(N \to N) = 1$$

which represents the probability that the population (size = N) including i mutants (fitness relative to resident = r) at generation t changes to the population including j mutants at generation $t + 1$.

We define $\tilde{v}_i$ as the probability that no successful secondary (final) genotype with relative swarm fitness a (probability of fixation = $U(a)$) is produced starting from the state where i swarms with intermediate genotype are present. For each possible state i, $\tilde{v}_i$ is given by:

$$\tilde{v}_0 = 1$$

$$\tilde{v}_i = (1-\omega)^i \sum_{j=0}^{N} \Pr(i \to j)\tilde{v}_j$$

$$\tilde{v}_N = 0,$$

where $\omega = \mu U(a)$ (mutation rate = μ). The second equation for $1 \le i \le N - 1$ can be rewritten as:

$$0 = (1-\omega)^i \sum_{j=0}^{N} \Pr(i \to j)\tilde{v}_j - \tilde{v}_i$$

$$= (1-\omega)^i \sum_{j=0}^{i-1} \Pr(i \to j)\tilde{v}_j + \left[(1-\omega)^i \Pr(i \to i) - 1\right]\tilde{v}_i + (1-\omega)^i \sum_{j=i+1}^{N} \Pr(i \to j)\tilde{v}_j$$

Then, $\tilde{v}_i$ can be written in matrix form as $\mathbf{A}\tilde{\mathbf{v}} = \mathbf{x}$ where $\mathbf{A}$, $\tilde{\mathbf{v}}$ and $\mathbf{x}$ are given by:

$$\mathbf{A} = \begin{pmatrix} 1 & 0 & \cdots & \cdots & 0 \\ (1-\omega)^1\Pr(1 \to 0) & (1-\omega)^1\Pr(1 \to 1) - 1 & (1-\omega)^1\Pr(1 \to 2) & \cdots & \cdots \\ (1-\omega)^2\Pr(2 \to 0) & (1-\omega)^2\Pr(2 \to 1) & (1-\omega)^2\Pr(2 \to 2) - 1 & \cdots & \cdots \\ \vdots & \vdots & \vdots & \ddots & \cdots \\ 0 & 0 & \cdots & \cdots & 1 \end{pmatrix}$$

$$\tilde{\mathbf{v}} = \begin{pmatrix} \tilde{v}_0 \\ \tilde{v}_1 \\ \vdots \\ \tilde{v}_N \end{pmatrix}$$

$$\mathbf{x} = \begin{pmatrix} 1 \\ 0 \\ \vdots \\ 0 \end{pmatrix}$$

Finally, we obtain v_1 from $\tilde{v}_1$ and the probability of fixation of the intermediate genotype $U(r)$ (1) as:

$$\begin{aligned} v_1 &= \tilde{v}_1(1 - U(r)) \\ &= \tilde{v}_1\left(1 - \frac{1 - e^{-2(r-1)}}{1 - e^{-2N(r-1)}}\right) \end{aligned}$$

In the case of the neutral intermediate, $U(r) = 1/N$.

Relative swarm fitness. Among the parameters of the population genetic model of stochastic tunneling, the relative fitness values of the genotypes {1,0,1;0} (r_0; neutral intermediate), {1,0,0;1} (r_-; inferior intermediate), and {1,0,1;1} (a; final), compared to the original genotype {1,0,0;0}, had to be estimated from simulations. The relative swarm fitness values were obtained from data of evolutionary simulations as follows: First, we collected a set of mean fitness values of the genotypes {1,0,0;0}, {1,0,1;0}, {1,0,0;1}, and {1,0,1;1} at each generation over the 50 evolutionary simulations. Then we resampled (with replacement) 50 quadruplets of mean fitness values (original, neutral intermediate, inferior intermediate, and final genotypes, respectively) from the above set and obtained 50 triplets of relative swarm fitness compared to the original genotype (r_0, r_-, and a).

Waiting time comparisons. Each of the 50 triplets of relative fitness values (r_0, r_-, and a), together with the population genetic model of stochastic tunneling described above, yielded a pair of analytical estimates of waiting time that assumed the evolutionary paths with, respectively, neutral and inferior intermediates. We compared the pairs of estimates and regarded the path with the shorter waiting time as the realized one in an evolving population. We repeated the procedure (i.e., resampling 50 quadruplets) 1000 times to obtain the distribution of the frequency of evolutionary runs (out of 50) that had a path with the neutral intermediate genotype {1,0,1;0}.

Supplementary Note 2. Evolutionary trajectories under different genetic coding

We made a simplified assumption about the genotype–phenotype mapping of robotic traits, in contrast to a standard approach taken by evolutionary robotics studies (e.g., 2, 3) such as a neural network with synaptic links evolving by genetic algorithm. In evolutionary swarm robotics, complex outcomes are derived from two hierarchical sources: the complexity of genotype–phenotype mapping attributed to each agent and the complexity of interactions between agents. Our approach separates these two sources, making the former simple and the latter intact. The simplified assumption of genetic architectures will complement the standard approach by fostering a deeper understanding of underlying evolutionary forces that lead to complex adaptive systems.

Although simple, our genotype–phenotype mapping enables biological realism of the phenotypes. First, a biologically realistic interpretation of the selectively neutral allele $b_3 = 1$ in the multilocus genotype $\{b_1,b_2,b_3;p\} = \{1,0,1,0\}$ (i.e., the behavior presuming pheromone communication in its absence) could be that the trait b_3 involves the behavior that is specifically released when colliding with the food-laden agent (with state S_2). The difference between $b_1 = 1$ and $b_3 = 1$ is that the latter requires a cognitive ability to assess collision partners (i.e., with or without food).

We can also consider different genetic coding of the three behavioral traits (b_1–b_3) to treat correlated phenotypes. Because the priority-giving behavior is phenotypically the same for b_1 to b_3, it is reasonable to consider their pleiotropic origin. First, we assume that the priority-giving behavior is regulated irrespective of the three internal states (S_1–S_3) of the robots. This means that $b_2 = b_3 = 1$ is a pleiotropic byproduct of $b_1 = 1$. Each evolutionary simulation run continued until the population reached the genotype with the highest swarm fitness among possible genotypes. All evolutionary simulations (50 replicates) resulted in the genealogy undergoing $\{0,0,0;0\}\rightarrow\{1,1,1;0\}\rightarrow\{1,1,1;1\}$, indicating that the regulatory mechanism ($b_1 = b_2 = b_3 = 1$) predated the pheromone detection ability ($p = 1$). This result is easily explained by a comparison of swarm fitness; that is, the swarm fitness of the genotype $\{1,1,1;0\}$ is much greater than that of the genotype $\{0,0,0;0\}$ than is that of the genotype $\{0,0,0;1\}$ (Fig. 2).

Second, we assume that the priority-giving behavior is regulated in relation to the food load of the robots. State S_2 (carrying food and recruiting) comes with food load, while states S_1 (searching) and S_3 (being recruited) do not. Therefore, we modeled so that only $b_3 = 1$ is a pleiotropic byproduct of $b_1 = 1$. All evolutionary simulations (50 replicates) resulted in the genealogy undergoing $\{0,0,0;0\}\rightarrow\{1,0,1;0\}\rightarrow\{1,0,1;1\}$, indicating that the regulatory behavior ($b_1 = b_3 = 1$) predated the pheromone detection ability ($p = 1$). As shown in Fig. 2, the swarm fitness of the genotype $\{1,0,1;0\}$ is greater than that of the genotype $\{0,0,0;1\}$.

These alternatives for genetic coding assume that the behaviors on the foraging trail ($b_2 = 1$ and $b_2 = b_3 = 1$) are a byproduct caused by the same genetic control as $b_1 = 1$. Since $b_1 = 1$ has an adaptive function in the absence of pheromone communication by improving the swarm fitness (Fig. 2), pleiotropically induced $b_2 = b_3 = 1$ (in the first case) and $b_3 = 1$ (in the second case) can be regarded as a case of spandrel (4), that is, a neutral byproduct of previous adaptation in other contexts (see Discussion). The above analyses confirmed the evolutionary precedence of regulatory behavior irrespective of genetic coding of phenotypes or of the number of behavioral genes compared to the pheromone-responsiveness gene.

Supplementary References

1. Proulx, S. R. The rate of multi-step evolution in Moran and Wright-Fisher populations. *Theor. Popul. Biol.* **80**, 197–207 (2011). DOI:10.1016/j.tpb.2011.07.003
2. Floreano, D. & Mattiussi, C. *Bio-Inspired Artificial Intelligence: Theories, Methods, and Technologies* (MIT Press, Cambridge, MA, 2008).
3. Doncieux, S., Bredeche, N., Mouret, J. B. & Eiben, A. E. G. Evolutionary robotics: what, why, and where to. *Front. Robot. AI* **2**, 4 (2015). DOI:10.3389/frobt.2015.00004
4. Gould, S. J. & Lewontin, R. C. The spandrels of San Marco and the Panglossian paradigm: a critique of the adaptationist programme. *Proc. R. Soc. Lond. B* **205**, 581–598 (1979). DOI:10.1098/rspb.1979.0086
