# Supplementary material for: Regulatory mechanism predates the evolution of self-organizing capacity in simulated ant-like robots
Source: Commun Biol. 2019 Jan 18;2:25. doi: 10.1038/s42003-018-0276-3 (PMC6338667; doi:10.1038/s42003-018-0276-3)
Supplement: Supplementary file 2 — Description of Additional Supplementary Files [file 42003_2018_276_MOESM2_ESM.docx]

**Description of Additional Supplementary Files**

**File Name**: Supplementary Movie 1

**Description**: Group foraging behavior performed by swarms with the genotypes {1,0,0;0} (original), {1,0,1;0} (neutral intermediate), {1,0,0;1} (inferior intermediate), and {1,0,1;1} (final).
